# Stabilizing σ-hole Dimethyl Interactions

**DOI:** 10.1021/acs.cgd.3c00347

**Published:** 2023-06-22

**Authors:** Noushin Keshtkar, Oliver Loveday, Víctor Polo, Jorge Echeverría

**Affiliations:** † Departamento de Química Física, Pedro Cerbuna 12, 50009 Zaragoza, Spain; ‡ Departament de Química Inorgànica i Orgànica and IQTC-UB, Universitat de Barcelona, Martí i Franquès 1-11, 08028 Barcelona, Spain; § Instituto de Biocomputación y Física de Sistemas Complejos (BIFI), Universidad de Zaragoza, 50009 Zaragoza, Spain; ∥ Departamento de Química Inorgánica and Instituto de Síntesis Química y Catálisis Homogénea (ISQCH), CSIC-Universidad de Zaragoza, Pedro Cerbuna 12, 50009 Zaragoza, Spain

## Abstract

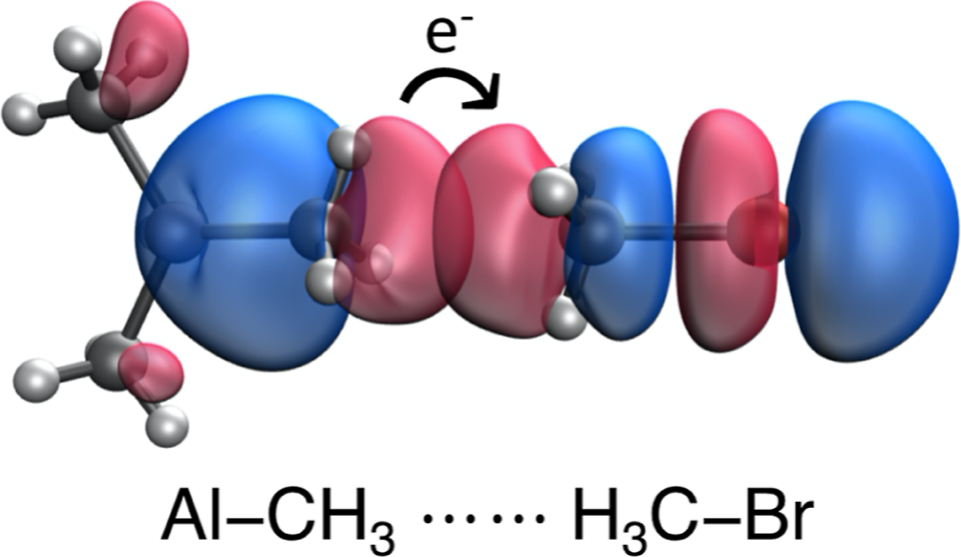

Methyl groups bound to electronegative atoms, such as N or O, are recognized to participate in tetrel bonding as Lewis acids. On the other hand, the capability of methyl groups bound to electropositive atoms, such as B or Al, to act as Lewis bases has been recently reported. Herein, we analyze the combination of these two behaviors to establish attractive methyl···methyl interactions. We have explored the Cambridge Structural Database to find experimental examples of these dimethyl-bound systems, finding a significant degree of directionality in the relative disposition of the two methyl groups. Moreover, we have carried out a comprehensive computational analysis at the DFT level of the dimethyl interactions, including the natural bond orbital, energy decomposition analysis, and topological analysis of the electron density (QTAIM and NCI). The dimethyl interaction is characterized as weak yet attractive and based on electrostatics, with a non-negligible contribution from orbital charge transfer and polarization.

## Abstract 2

Methyl groups, depending on the atom they are bound to, can behave as both Lewis acids and bases to engage in highly directional and stabilizing dimethyl interactions.

## Introduction

Methyl groups play an important role in chemistry because they act as functional groups in organic synthesis, contributing to molecular structure and reactivity; they are present in many naturally occurring molecules, including DNA and hormones; they participate in the regulation of gene expression through epigenetic modifications; and they also have an impact on the physical properties of molecules, such as solubility and boiling point.

Methyl groups in hydrocarbon chains participate in several types of noncovalent interactions, such as, for instance, forming hydrogen bonds with nitrogen and oxygen atoms (C–H···O/N).^1^ In aqueous solutions, methyl groups can participate in hydrophobic interactions by avoiding contact with water.^2^ When a methyl group is directly bound to an electronegative atom (e.g., nitrogen, oxygen, or chlorine) the electron density is attracted by the later and the electron-deficient carbon atom can behave as a Lewis acid giving place to the so-called tetrel bonding.^3−6^ On the other hand, if the methyl group is bound to an electropositive atom (e.g., lithium, boron, or aluminium), the carbon atoms become electron-rich and can act as a Lewis base in many different types of known noncovalent interactions. This latter behavior has been recently reported in hydrogen bonds,^7^ σ-^8^ and π-hole^9^ interactions, short methyl-alkali metal contacts in aluminates,^10^ and in metal–ligand interactions in proteins.^11^

The idea of group 14 atoms to act as Lewis bases has been explored in the last few years, mainly by means of computational tools.^12−18^ In a recent report, Scheiner demonstrated the ability of a tetrel atom to serve as an electron donor to another tetrel atom within the context of a tetrel bond.^19^ Herein, we analyze a hypothetical methyl–methyl interaction in which each of the partners acts as the Lewis base and acid, respectively. First, we searched the Cambridge Structural Database^20^ (CSD) for possible methyl···methyl short contacts with some degree of directionality. Then, a comprehensive computational analysis has been performed on selected systems to unveil the nature and strength of these interactions.

## Results and Discussion

### Structural Analysis

**Scheme 1:**
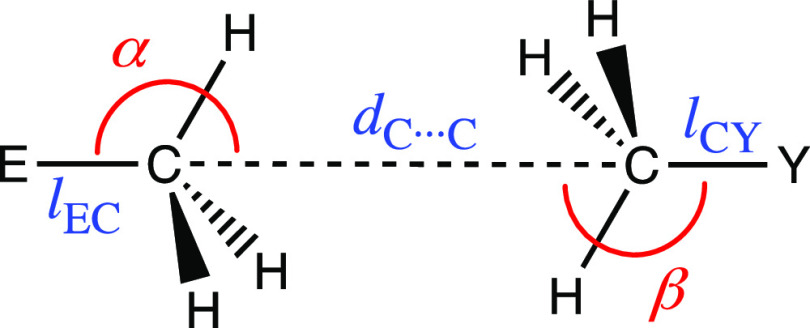
Main Geometrical Parameters Associated with methyl···methyl Short Contacts Studied in This Work

The main structural parameters associated with methyl···methyl interactions are summarized in Scheme 1. We have searched in the CSD for E–C(H_3_)···CH_3_–Y (E = B–Tl, Si–Pb; Y = N–Bi, O–Po, F–At) contacts shorter than the sum of the van der Waals radii +0.1 Å (*d*_C···C_ < 3.64 Å) and two angles, defined as E–C···C (α) and Y–C···C (β) associated with the directionality of electron-rich and electron-deficient carbon atoms, respectively.

**Figure 1:**
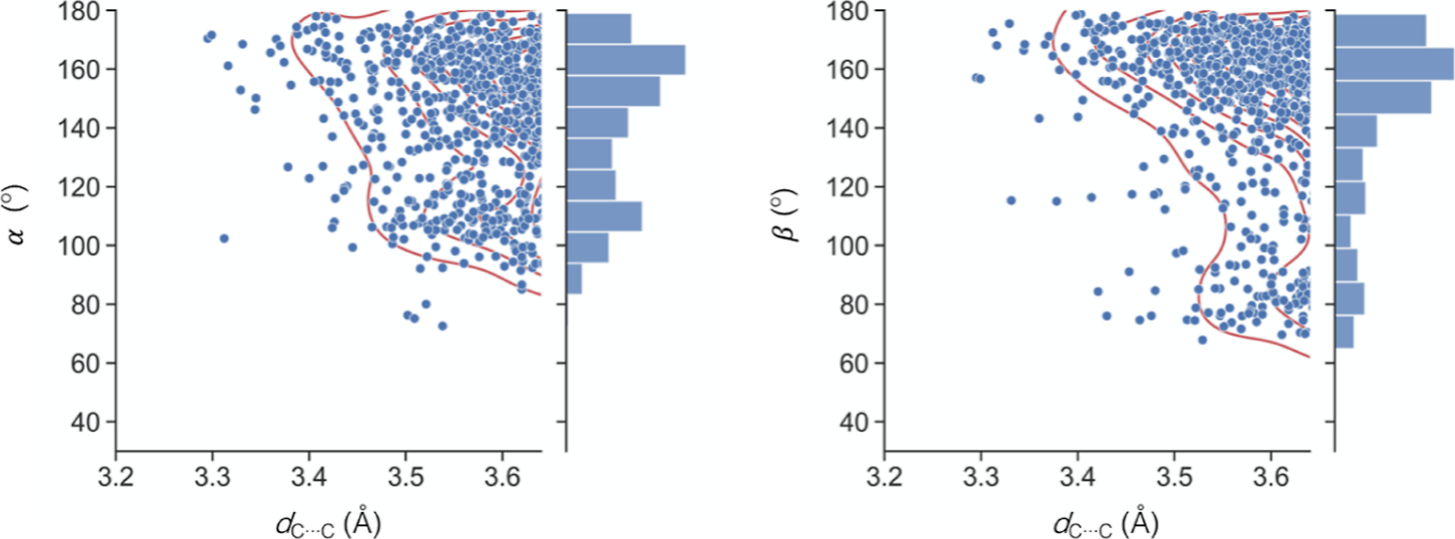
Dependence of α (left) and β (right) angles with the intermolecular C···C distance (< sum of vdW radii +0.1 Å) for short E–CH_3_···CH_3_–Y (E = B–Tl, Si–Pb; Y = N–Bi, O–Po, F–At) contacts found in the CSD.

The C···C distances are somewhat long, with penetration indexes^21^ (*p*_C···C_) smaller than 12%, corresponding to distances larger than 3.3 Å. However, we have found a significant degree of directionality in the relative orientations of the two involved methyl groups. In general, for both α and β, the shorter the C···C distance, the closer the interacting angle to 180° (Figure 1). This interaction topology should maximize an attractive electrostatic interaction since we know from previous reports that the molecular electrostatic potential at the carbon atom in the E–CH_3_ moiety is negative while that in the Y–CH_3_ is positive. Although angles between 150 and 180° are more abundant in the light of the histograms of Figure 1, there is a smaller shoulder at angles between 80 and 120°, i.e., at the extension of one of the H–C bonds of the methyl group.

### Optimized Geometries and Interaction Energies

Next, we have selected a set of E–CH_3_···H_3_C–Y model systems to analyze in detail the dimethyl interaction, where Y can be −CH_3_, −N(CH_3_)_2_, −OCH_3_ and −Br, and E can be (CH_3_)_3_–Si–, (CH_3_)_3_–Sn–, and (CH_3_)_2_–Al–. The combination of these carbon-based Lewis acids and bases gives place to adducts **1**–**12**, as shown in Table 1. After full geometry optimization, all adducts show a linear geometry with a methyl–methyl contact shorter than twice the carbon van der Waals radii and an alternate disposition of the hydrogen atoms. Both α and β angles display values very close to 180° in all cases. The carbon···carbon distances range from 3.374 to 3.563 Å, which involves very small penetrations of the corresponding van der Waals crusts (*p*_C···C_ from 8.2 to −1.1%). For the different E groups, carbon···carbon distances follow the decreasing trend Al > Si > Sn, while for Y, the trend is CH_3_ > N(CH_3_)_2_ > OCH_3_ > Br. It is known that methyl groups can engage in relatively strong C···H and dihydrogen H···H interactions.^22,23^ However, in the adducts studied here, all H···H and C···H distances are longer than the sum of the van der Waals radii, showing negative penetration indexes (*p*_C···H_ and *p*_H···H_ smaller than −10 and −24%, respectively), which makes us expect very little contribution from these particular interactions to the overall stability of the adducts. In the next section, a topological analysis of the electron density should shed light on the role of any specific short contact.

**Table 1:** Geometrical Parameters and Interaction Energies of Fully Optimized E–CH_3_···H_3_C–Y (**1**–**12**) Adducts at the M06-2X/def2-TZVPD Level

adduct	E	Y	*d*_C···C_ (Å)	α (deg)	β (deg)	Δ*E*_int_ (kcal/mol)
**1**	(CH_3_)_3_–Si–	–CH_3_	3.505	179.4	179.4	–0.51
**2**	(CH_3_)_3_–Sn–	–CH_3_	3.509	179.8	179.5	–0.52
**3**	(CH_3_)_2_–Al–	–CH_3_	3.563	177.0	179.6	–0.49
**4**	(CH_3_)_3_–Si–	–N(CH_3_)_2_	3.445	179.3	178.9	–0.69
**5**	(CH_3_)_3_–Sn–	–N(CH_3_)_2_	3.444	179.7	178.8	–0.71
**6**	(CH_3_)_2_–Al–	–N(CH_3_)_2_	3.471	177.8	178.1	–0.73
**7**	(CH_3_)_3_–Si–	–OCH_3_	3.389	179.9	178.0	–0.84
**8**	(CH_3_)_3_–Sn–	–OCH_3_	3.386	179.7	178.0	–0.88
**9**	(CH_3_)_2_–Al–	–OCH_3_	3.409	178.1	177.2	–0.93
**10**	(CH_3_)_3_–Si–	–Br	3.382	179.3	179.7	–1.00
**11**	(CH_3_)_3_–Sn–	–Br	3.374	179.6	179.9	–1.08
**12**	(CH_3_)_2_–Al–	–Br	3.389	178.8	179.7	–1.19

**Figure 2:**
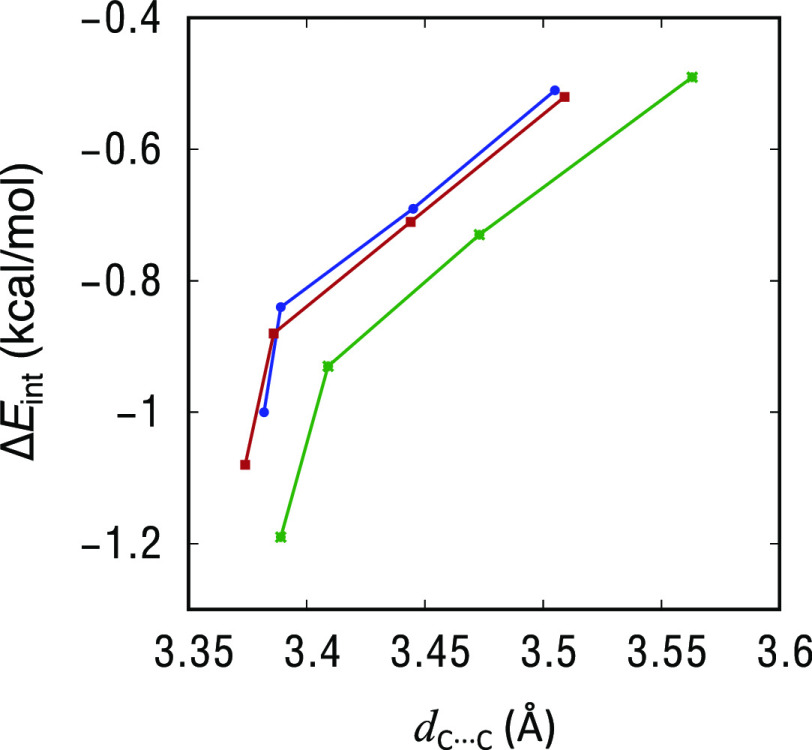
Dependence of the M06-2X interaction energy with the *d*_C···C_ intermolecular contact distances for adducts **1**–**12**. (Blue = Si; red = Sn; and green = Al).

The calculated interaction energies show attractive interactions in all cases, and they correlate with the intermolecular C···C distance; the stronger the interaction, the shorter the C···C distance (Figure 2). The strength of the interaction for the different Y groups follows the same trend as the carbon···carbon distances, while if we look at E, it is strongest for Al. The interaction energy values are small, between −0.5 and −1.2 kcal/mol, in good agreement with the relatively long carbon···carbon distances obtained in the geometry optimizations. Due to the small values of the interaction energy and in order to check whether the interaction is truly attractive, we have reoptimized systems **1**–**12** at the MP2 level (see complete results in Table S1 in the Supporting Information). The C···C interatomic distances are longer (∼0.3 Å), and the interaction energies are up to 21.5% smaller than those at the DFT level. Remarkably, the same dependence of the C···C distance with the interaction energy observed in Figure 2 is observed at the MP2 level (Figure S1 in the Supporting Information). An energy decomposition analysis of adducts **1**–**12** disclosed that the combination of electrostatics and dispersion is able to overcome Pauli exchange repulsion in all cases, with significant contributions of polarization and charge transfer, especially for the stronger interactions (see complete results in Table S2 in the Supporting Information).

### Topological Analysis of the Electron Density

**Figure 3:**
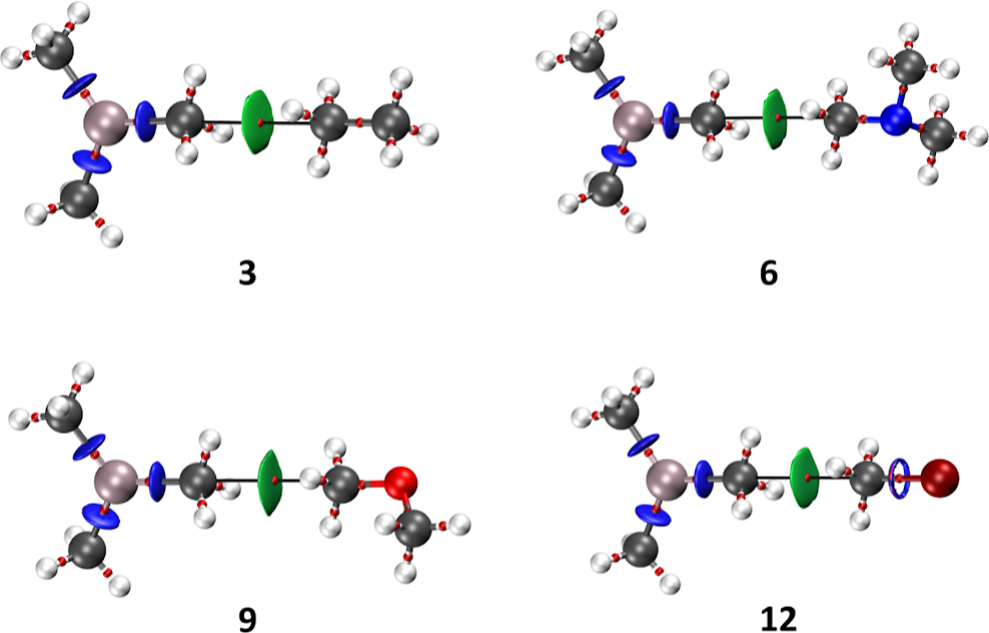
Combined NCI and QTAIM plots for systems **3**, **6**, **9**, and **12**.

**Figure 4:**
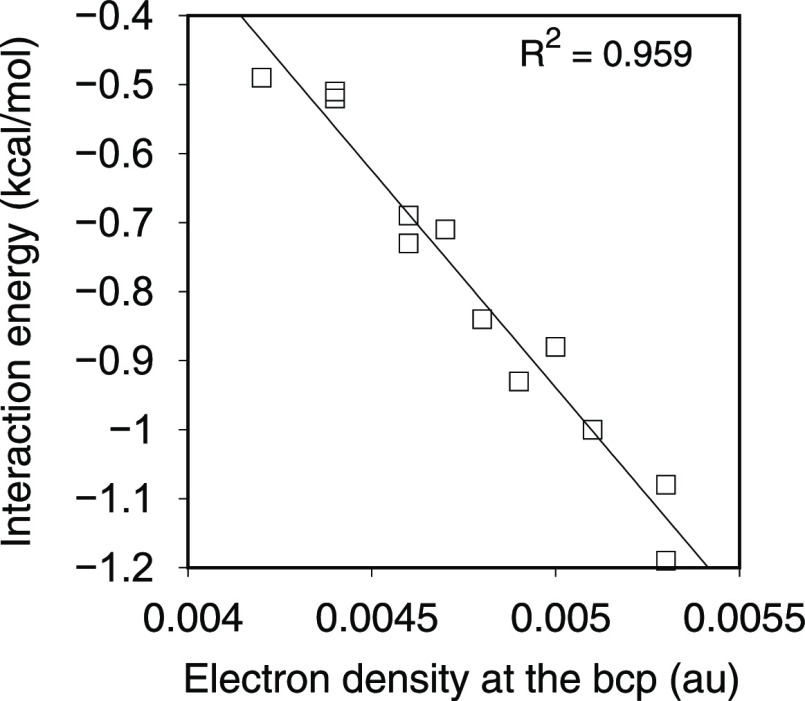
Linear dependence of the interaction energy with the value of the electron density at the bcp for the adducts **1**–**12**.

The study of the electron density and its derivatives usually gives useful information on the nature of an interaction, while the analysis of the reduced density gradient helps identify the specific regions of intermolecular attractive interaction. We have applied QTAIM and NCI methods to adducts **1**–**12**. The results for the adducts in which the Lewis base is Al(CH_3_)_3_ (**3**, **6**, **9**, and **12**) are shown in Figure 3. It can be observed that, in the four systems, there is only one bond path (bp) with its corresponding bond critical point (bcp) between the two molecules. Remarkably, the bp clearly connects the two carbon atoms at the methyl groups. Moreover, the green NCI isosurfaces are associated with regions of weak attraction between the two methyl groups. If we look at the values of relevant QTAIM parameters at the bcp, we can conclude that the interaction is weak and of a closed-shell nature (complete QTAIM results are in Table S3 at the Supporting Information). We have also found a nice linear correlation between the interaction energy and the value of the electron density at the bcp for adducts **1**–**12** (Figure 4).

### Orbital Interactions

**Figure 5:**
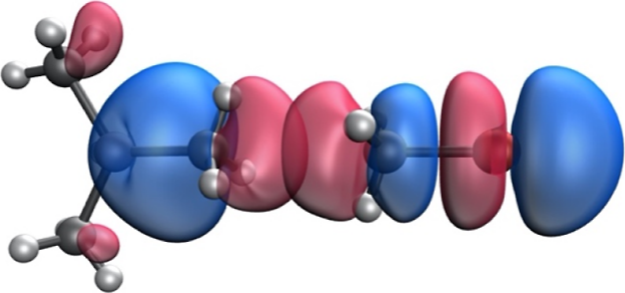
Donor and acceptor natural bond orbitals involved in the σ_Al__–__C_ → σ*_C–Br_ charge transfer process in adduct **12**.

We observed in our EDA results that charge transfer plays a significant role in the stabilization of dimethyl interacting systems compared with the other energy terms, so we decided to perform a natural bond orbital (NBO) analysis of the Al-containing adducts (**3**, **6**, **9,** and **12**; those showing a stronger interaction) to unveil the orbitals involved in the process. The charge transfer processes along with the corresponding second-order perturbation energies are summarized in Table 2. The main donor orbitals are σ bonding orbitals associated with the Al–C bond, while the acceptor orbitals are σ* orbitals associated with the C–Y (Y = C, N, O, and Br) bonds (Figure 5). There is also charge transfer from the three σ_C–H_ orbitals of the electron-rich methyl groups into the σ*_C–Y_ orbital of the other molecule. Accordingly, the whole methyl group acts as a donor via the available electron density from C and the C–H bonds.

**Table 2:** NBO Second Order Perturbation Energies (*E*^(2)^) for the Donor–Acceptor Orbital Interactions in Adducts **3**, **6**, **9**, and **12**

adduct	donor	acceptor	*E*^(2)^ (kcal/mol)
**3**	σ_Al–C_	σ*_C–C_	0.10
**6**	σ_Al–C_	σ*_C–N_	0.15
	σ_C–H_	σ*_C–N_	0.07/0.07/0.06
**9**	σ_Al–C_	σ*_C–O_	0.16
	σ_C–H_	σ*_C–O_	0.06/0.06/0.06
**12**	σ_Al–C_	σ*_C–Br_	0.22
	σ_C–H_	σ*_C–Br_	0.13/0.12/0.12

**Figure 6:**
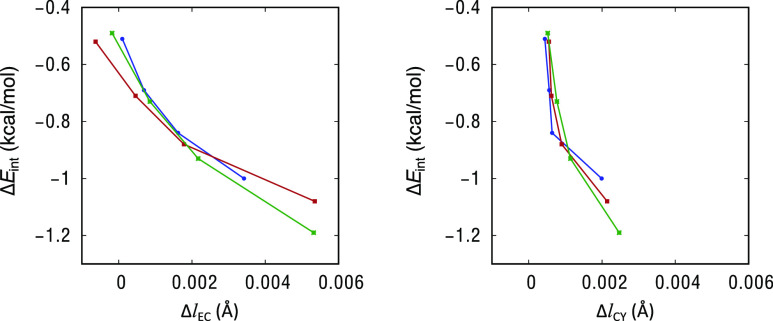
Changes in E–C (left) and C–Y (right) bond lengths upon interaction for adducts **1**–**12**. (Blue = Si; red = Sn; and green = Al). Results from M06-2X/def2-TZVPD calculations.

Charge transfer processes are usually associated with changes in the bond lengths of the involved moieties. In the present case, upon interaction, we observe an elongation of the E–C bond associated with the depopulation of a bonding orbital (σ_E–C_) and an elongation of the C–Y bond associated with the population of an antibonding orbital (σ*_C–Y_), as shown in Figure 6. The changes are very small, especially in the C–Y bond, but it has to be considered that the charge transfer processes are scarcely energetic.

## Conclusions

The capability of two methyl groups to attractively interact with each other has been investigated herein. We have observed that an electron-rich methyl group (bound to atoms less electronegative than carbon) and an electron poor methyl group (bound to atoms more electronegative than carbon) are able to establish a stabilizing dimethyl interaction. An exploration of the CSD has disclosed several existing crystal structures with short methyl···methyl contacts. The carbon···carbon distances are within the sum of the van der Waals radii with small penetration indices (<12%), displaying a marked preference for linearity in the E–C···C–Y framework as the interatomic distance shortens. A set of selected model systems has been selected and investigated at the DFT level, showing clear linearity after geometry optimization. The calculated interaction energies are small, ranging from −0.5 to −1.2 kcal/mol and being strongest for E = Al. The analysis of the topology of the electron density has shown that the interaction is localized between the two carbon atoms without contribution from C···H or H···H interactions, in good agreement with the negative penetration indices obtained for these pairs of atoms in the model systems. Finally, an NBO analysis has been performed to unveil charge transfer processes from bonding to antibonding carbon-based orbitals (σ_E–C_ → σ*_C–Y_). We believe that the results presented here will contribute to understanding the rich supramolecular chemistry of methyl groups and exploiting it in crystal and material design.

## Computational Details

Structural searches were carried out in the CSD, version 5.41 (November 2019) with 3 updates.^20^ Crystal structures with 3D coordinates defined, non-disordered, with no errors, not polymeric, and with R lower than 0.05 were considered. CSD identifiers are given throughout the manuscript as six-letter refcodes (e.g., ABCDEF). For the analysis of interatomic distances, we used the recently proposed penetration index. The penetration index *p*_AB_ indicates the degree of interpenetration of the van der Waals crusts of atoms A and B from 0% (canonical vdW contact) to 100% (canonical bond distance) and is defined as *p*_AB_ = 100·(*v*_A_ + *v*_B_ – *d*_AB_)/(*v*_A_ + *v*_B_ – *r*_A_ – *r*_B_), where *v* is the van der Waals radius, and *r* is the covalent radius of a given atom. For computing *p*, we used standard sets of van der Waals^24^ and covalent^25^ radii. Further details on the use of penetration indexes and their applications can be found in a recent publication.^21^

DFT and NBO calculations were performed with the M06-2X functional and the def2-TZVPD basis sets for all atoms. We chose the M06-2X functional because of its good performance with noncovalent interactions in previous benchmark reports.^26,27^ All adducts were fully optimized and characterized as true minima of the corresponding potential energy surfaces by means of vibrational analysis. All systems were further optimized at the MP2/def2-TZVPD level to test the validity of DFT calculations. Interaction energies were calculated via the supermolecule approach and corrected for the BSSE by means of the counterpoise method^28^ with Gaussian16.^29^ For the decomposition of the interaction energy, we used the second-generation ALMO-EDA method implemented in Q-Chem5.3.^30^ QTAIM and NCI topological analyses of the electron density were carried out with MultiWfn 3.7 on the DFT wavefunction.^31^

## Supporting Information Available

Complete EDA and QTAIM results, MP2 optimizations, and a text file of all computed molecule Cartesian coordinates in a format for convenient visualization (PDF)
