# Supplementary material for: Stabilizing σ-hole Dimethyl Interactions
Source: Cryst Growth Des. 2023 Jun 22;23(7):5112–6. doi: 10.1021/acs.cgd.3c00347 (PMC10327473; doi:10.1021/acs.cgd.3c00347)
Supplement: Supplementary file 1 — cg3c00347_si_001.pdf [file cg3c00347_si_001.pdf]

Stabilizing σ-hole dimethyl interactions

Noushin Keshtkar,[a] Oliver Loveday,[b] Víctor Polo[a,c]* and Jorge Echeverría[d]*

a) Departamento de Química Física, Pedro Cerbuna 12, 50009 Zaragoza (Spain).

b) Departament de Química Inorgànica i Orgànica and IQTC-UB, Universitat de Barcelona, Martí i Franquès 1-11, 08028 Barcelona (Spain).

c) Instituto de Biocomputación y Física de Sistemas Complejos (BIFI), Universidad de Zaragoza, 50009 Zaragoza (Spain).

d) Departamento de Química Inorgánica and Instituto de Síntesis Química y Catálisis Homogénea (ISQCH), CSIC-Universidad de Zaragoza, Pedro Cerbuna 12, 50009 Zaragoza (Spain).

e-mail: jorge.echeverria@unizar.es

SUPPORTING INFORMATION

Table S1. Geometrical parameters and BSSE-corrected interaction energies of fully optimized E-$CH_3\cdots H_3C$-Y (**1** – **12**) adducts at the MP2/def2-TZVPD level.

Adduct	E	Y	$d_{C\cdots C}$ (Å)	α (°)	β (°)	ΔE_{BSSE} (kcal/mol)
**1**	$(CH_3)_3$-Si-	-CH_3	3.815	180.0	180	-0.49
**2**	$(CH_3)_3$-Sn-	-CH_3	3.822	179.7	179.5	-0.49
**3**	$(CH_3)_2$-Al-	-CH_3	3.892	176.9	179.6	-0.4
**4**	$(CH_3)_3$-Si-	-$N(CH_3)_2$	3.696	179.2	178.9	-0.69
**5**	$(CH_3)_3$-Sn-	-$N(CH_3)_2$	3.696	179.6	178.7	-0.71
**6**	$(CH_3)_2$-Al-	-$N(CH_3)_2$	3.75	178.1	178.7	-0.65
**7**	$(CH_3)_3$-Si-	-OCH_3	3.625	179.9	177.9	-0.72
**8**	$(CH_3)_3$-Sn-	-OCH_3	3.655	179.6	177.9	-0.74
**9**	$(CH_3)_2$-Al-	-OCH_3	3.695	178.1	177.2	-0.73
**10**	$(CH_3)_3$-Si-	-Br	3.621	179.3	179.7	-0.87
**11**	$(CH_3)_3$-Sn-	-Br	3.61	179.5	179.9	-0.92
**12**	$(CH_3)_2$-Al-	-Br	3.647	178.7	179.6	-0.95

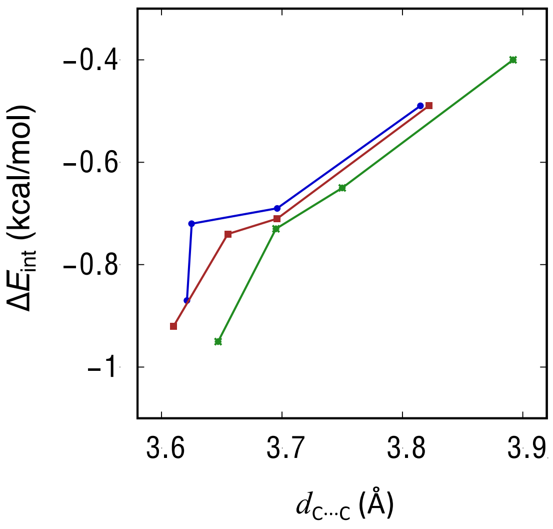

Figure S1. Dependence of the MP2 interaction energy with the $d_{C\cdots C}$ intermolecular contact distances for adducts **1** – **12**. (Blue = Si; red = Sn; green = Al).

Table S2. Energy decomposition analysis (EDA) of the E-CH_3···H_3C-Y (**1** – **12**) adducts at the M06-2X/def2-TZVPD level. The ALMO-EDA scheme decomposes the interaction energy into terms that are chemically meaningful, namely polarization (ΔE_{POL}), charge transfer (ΔE_{CT}) and a frozen energy (ΔE_{FRZ}) that is composed of electrostatics, Pauli repulsion and dispersion. All energies are given in kcal/mol.

Adduct	ΔE_{FRZ}	ΔE_{POL}	ΔE_{CT}	ΔE_{INT}
**1**	-0.27	-0.06	-0.18	-0.51
**2**	-0.26	-0.07	-0.19	-0.52
**3**	-0.27	-0.05	-0.17	-0.49
**4**	-0.37	-0.09	-0.23	-0.69
**5**	-0.37	-0.10	-0.24	-0.71
**6**	-0.40	-0.09	-0.23	-0.73
**7**	-0.44	-0.12	-0.27	-0.84
**8**	-0.45	-0.14	-0.28	-0.88
**9**	-0.51	-0.13	-0.28	-0.93
**10**	-0.45	-0.21	-0.34	-1.00
**11**	-0.48	-0.23	-0.37	-1.08
**12**	-0.59	-0.23	-0.37	-1.19

Table S3. Values of different QTAIM parameters, namely the electron density (**ρ**), the Laplacian of the electron density (**$\nabla^2\rho$**), the electronic total energy density (**H**) and the delocalization index between the two C atoms involved in the interaction (**DI (C, C)**), at the BCPs for adducts **1** – **12** computed at the M06-2X/def2-TZVPD. All values are given in a.u.

**Adduct**	**ρ**	**$\nabla^2\rho$**	**H**	**DI (C, C)**
**1**	0.0044	0.0167	0.0008	0.0142
**2**	0.0044	0.0163	0.0008	0.0141
**3**	0.0042	0.0154	0.0008	0.0141
**4**	0.0046	0.0183	0.0009	0.0147
**5**	0.0047	0.0181	0.0008	0.0148
**6**	0.0046	0.0178	0.0009	0.0154

**7**	0.0048	0.0200	0.0010	0.0160
**8**	0.050	0.0198	0.0009	0.0161
**9**	0.0049	0.0197	0.0010	0.0170
**10**	0.0051	0.0193	0.0009	0.0185
**11**	0.0053	0.0192	0.0008	0.0190
**12**	0.0053	0.0194	0.0009	0.0202

Table S4. Cartesian coordinates of all optimized systems characterized as real minima of the corresponding potential energy surfaces.

Adduct 1 / (CH3)3-Si-CH3⋯H3C-CH3 / M06-2X

Si	0.001897	-0.002056	-0.084612
C	0.007364	-0.014218	1.790207
H	1.026770	-0.018750	2.181954
H	-0.498739	0.865915	2.192452
H	-0.502224	-0.897679	2.180626
C	-1.767211	0.004921	-0.705317
C	0.886290	1.530889	-0.703234
C	0.881119	-1.529970	-0.722788
H	0.898073	1.562773	-1.794597
H	0.394583	2.438537	-0.347565
H	1.921494	1.553666	-0.356903
H	0.892879	-1.548058	-1.814468
H	1.916213	-1.560655	-0.376719
H	0.386337	-2.440396	-0.378673
H	-1.799519	0.011926	-1.796717
H	-2.307880	-0.878787	-0.360565
H	-2.304945	0.885980	-0.349349
C	-0.006525	-0.006546	5.295307
C	-0.006995	0.011936	6.818536
H	1.009116	0.033024	7.213725
H	-0.529358	0.888591	7.202866
H	-0.501082	-0.871439	7.224233
H	0.515470	-0.882289	4.907905
H	-1.021652	-0.027817	4.897110
H	0.487012	0.875860	4.886558

Adduct 2 / (CH3)3-Sn-CH3⋯H3C-CH3 / M06-2X

Sn	0.000869	-0.000349	-0.176376
C	0.002950	-0.006644	1.982135
H	1.023113	-0.006599	2.366078
H	-0.507435	0.874822	2.370224
H	-0.505545	-0.891476	2.365006
C	-2.035003	0.004736	-0.895511
C	1.020209	1.762815	-0.893385

C	1.015157	-1.762206	-0.903669
H	1.030160	1.782458	-1.982986
H	0.521424	2.664419	-0.538443
H	2.051146	1.779114	-0.540413
H	1.023383	-1.776629	-1.993363
H	2.046574	-1.782567	-0.552324
H	0.514941	-2.664386	-0.552220
H	-2.055892	0.006928	-1.985135
H	-2.567573	-0.878620	-0.543792
H	-2.564242	0.888738	-0.540342
C	-0.004696	-0.006829	5.491262
C	-0.004215	0.004602	7.014671
H	1.012109	0.019531	7.409586
H	-0.522668	0.881522	7.403668
H	-0.501733	-0.878524	7.416751
H	0.513082	-0.882993	5.099326
H	-1.019941	-0.021724	5.093299
H	0.492314	0.875220	5.085921

Adduct 3 / (CH3)2-Al-CH3···H3C-CH3 / M06-2X

Al	-0.044343	0.748000	0.223751
C	-1.974984	0.462212	0.333706
H	-2.303856	0.072356	1.298250
H	-2.518870	1.393766	0.150236
H	-2.310736	-0.237271	-0.437863
C	0.749085	1.446098	-1.420977
C	1.093505	0.335837	1.759075
H	2.151380	0.534099	1.581600
H	0.787381	0.915984	2.634615
H	0.994038	-0.715847	2.043502
H	1.493383	0.749764	-1.818386
H	0.018171	1.637085	-2.207905
H	1.282893	2.381141	-1.227127
C	-5.509523	0.009293	0.360252
C	-7.020770	-0.181774	0.381019
H	-7.356565	-0.574078	1.341521
H	-7.541053	0.761131	0.209448
H	-7.341691	-0.881205	-0.391646
H	-4.986169	-0.931895	0.531913
H	-5.170355	0.401497	-0.599138
H	-5.185220	0.708207	1.131955

Adduct 4 / (CH3)3-Si-CH3···H3C-N(CH3)2 / M06-2X

C	1.789075	0.087008	-0.121511
Si	-0.079870	-0.010077	-0.000265
C	-0.790843	-0.472863	-1.672059
C	-0.556466	-1.309488	1.264238
C	-0.757627	1.655989	0.528519
C	5.224595	0.270129	-0.300802
N	6.669692	0.320711	-0.383773
H	2.097902	0.838451	-0.851453
H	2.232490	0.355596	0.839779

H	2.213932	-0.871043	-0.428741
H	-1.846192	1.629620	0.608494
H	-0.357928	1.951396	1.500690
H	-0.493958	2.433169	-0.191631
H	-1.879958	-0.537714	-1.630766
H	-0.528492	0.268928	-2.429055
H	-0.410734	-1.440401	-2.005978
H	-1.641572	-1.388202	1.355967
H	-0.172707	-2.291753	0.981386
H	-0.154479	-1.064312	2.249344
H	4.803770	0.014716	-1.273930
H	4.918514	-0.493768	0.414810
H	4.790205	1.233506	0.018792
C	7.094600	1.295831	-1.366753
C	7.249423	0.610084	0.911535
H	8.337499	0.607411	0.839713
H	6.930779	1.593401	1.299137
H	6.950115	-0.153527	1.630085
H	8.182318	1.294839	-1.443828
H	6.680220	1.042260	-2.342964
H	6.767175	2.317593	-1.106998

Adduct 5 / (CH3)3-Sn-CH3···H3C-N(CH3)2 / M06-2X

C	1.949958	0.095612	-0.120397
Sn	-0.202387	-0.022519	0.006742
C	-1.007975	-0.553464	-1.924529
C	-0.754641	-1.523714	1.456617
C	-0.991478	1.892673	0.615535
C	5.384281	0.285527	-0.304610
N	6.829206	0.336355	-0.391408
H	2.251231	0.850895	-0.846459
H	2.377883	0.361813	0.846236
H	2.369454	-0.862194	-0.428504
H	-2.078167	1.853494	0.687495
H	-0.592924	2.178667	1.588669
H	-0.722654	2.663896	-0.105980
H	-2.094858	-0.619524	-1.880438
H	-0.739349	0.194726	-2.669964
H	-0.618286	-1.517839	-2.249675
H	-1.838986	-1.599029	1.534993
H	-0.362841	-2.498357	1.166506
H	-0.355967	-1.271673	2.439044
H	4.960770	0.025679	-1.275368
H	5.079936	-0.475185	0.415081
H	4.950023	1.250019	0.011760
C	7.251255	1.307361	-1.379624
C	7.412029	0.631588	0.901143
H	8.499941	0.629137	0.826691
H	7.093866	1.616438	1.285293
H	7.114842	-0.129012	1.623759
H	8.338785	1.306532	-1.459500
H	6.834471	1.049466	-2.353679
H	6.924057	2.330122	-1.123447

Adduct 6 / (CH3)2-Al-CH3···H3C-N(CH3)2 / M06-2X

C	1.159610	0.022450	-0.101920
Al	3.11186	0.00775	0.00968
C	4.08562	1.68174	0.27490
C	4.08574	-1.68014	-0.14333
C	-2.30624	-0.08003	-0.26081
N	-3.74992	-0.11467	-0.37392
H	0.71840	-0.68320	0.60800
H	0.72125	1.00382	0.08658
H	0.82945	-0.30115	-1.09415
H	4.60911	-1.90533	0.79105
H	3.44366	-2.53122	-0.37445
H	4.85752	-1.62067	-0.91574
H	3.87115	2.38685	-0.53361
H	3.76662	2.17415	1.19821
H	5.16748	1.55092	0.32363
H	-1.89040	-1.06366	-0.48134
H	-1.89203	0.63278	-0.97447
H	-1.97250	0.21440	0.74950
C	-4.31359	-1.09350	0.53243
C	-4.31635	1.19603	-0.13192
H	-5.39896	1.16140	-0.25807
H	-4.09840	1.56135	0.88682
H	-3.90941	1.91276	-0.84584
H	-5.39626	-1.13373	0.40846
H	-3.90511	-2.08030	0.31245
H	-4.09501	-0.85655	1.58833

Adduct 7 / (CH3)3-Si-CH3···H3C-O(CH3) / M06-2X

C	1.814747	0.088131	-0.115597
Si	-0.054716	-0.007972	0.008210
C	-0.766750	-0.462756	-1.665199
C	-0.530705	-1.312273	1.267433
C	-0.729169	1.656917	0.544332
C	5.191642	0.263422	-0.336577
O	6.593094	0.298833	-0.395607
H	2.120507	0.840954	-0.845600
H	2.258601	0.354540	0.846153
H	2.237473	-0.870043	-0.425682
H	-1.817777	1.630940	0.624198
H	-0.329459	1.947740	1.517852
H	-0.465308	2.436966	-0.172623
H	-1.855949	-0.525917	-1.623070
H	-0.504196	0.281829	-2.419374
H	-0.388723	-1.429632	-2.003341
H	-1.615891	-1.389082	1.360105
H	-0.149600	-2.294042	0.979432
H	-0.127256	-1.072549	2.253234
H	4.757971	-0.002837	-1.308338
H	4.907202	-0.489362	0.395883
H	4.780669	1.233009	-0.029467

C	7.056766	1.246638	-1.319865
H	8.144397	1.215712	-1.309276
H	6.700975	1.020093	-2.332370
H	6.723688	2.256943	-1.052613

Adduct 8 / (CH3)3-Sn-CH3···H3C-O(CH3) / M06-2X

C	2.001469	0.098764	-0.117536
Sn	-0.151251	-0.018577	0.022298
C	-0.964856	-0.541844	-1.907508
C	-0.695415	-1.524004	1.470086
C	-0.932740	1.896608	0.639721
C	5.374399	0.282013	-0.355441
O	6.775694	0.320219	-0.422111
H	2.296205	0.855233	-0.845203
H	2.434197	0.363325	0.847466
H	2.417656	-0.859123	-0.430109
H	-2.019348	1.859714	0.714162
H	-0.531254	2.178553	1.612808
H	-0.664099	2.669458	-0.080120
H	-2.051905	-0.602569	-1.860011
H	-0.695156	0.206649	-2.652258
H	-0.581022	-1.507483	-2.235845
H	-1.779343	-1.597903	1.555099
H	-0.307004	-2.498130	1.173831
H	-0.290160	-1.276079	2.450824
H	4.936199	0.011979	-1.324053
H	5.095280	-0.469083	0.380761
H	4.963131	1.251646	-0.049064
C	7.232150	1.266253	-1.351569
H	8.319920	1.238075	-1.346924
H	6.871401	1.035923	-2.361481
H	6.898084	2.276585	-1.085589

Adduct 9 / (CH3)2-Al-CH3···H3C-O(CH3) / M06-2X

C	-1.993428	0.507236	0.153981
Al	-0.055698	0.757272	0.054538
C	1.023910	0.650380	1.679862
C	0.792775	1.115185	-1.669160
C	-5.373188	0.064870	0.217591
O	-6.754564	-0.177750	0.273620
H	-2.514864	1.416285	-0.162112
H	-2.320672	-0.282973	-0.528219
H	-2.346126	0.254082	1.155107
H	1.492996	0.314422	-1.925723
H	0.082228	1.208803	-2.491542
H	1.384901	2.033890	-1.630491
H	2.089440	0.803074	1.503209
H	0.697177	1.395329	2.411423
H	0.901651	-0.322878	2.164086
H	-5.083427	0.880335	0.891408
H	-4.861067	-0.844920	0.523808
H	-5.055387	0.327933	-0.798665

C	-7.501515	0.946543	-0.106879
H	-8.555254	0.683487	-0.039399
H	-7.301141	1.798274	0.554637
H	-7.271447	1.245521	-1.136880

Adduct 10 / (CH3)3-Si-CH3···H3C-Br / M06-2X

C	0.012663	0.000555	-0.084123
Si	0.002880	0.000296	1.793375
C	1.768435	-0.000003	2.421676
C	-0.884631	1.532154	2.408213
C	-0.884851	-1.531632	2.407541
C	-0.008053	0.000142	-3.466138
Br	-0.018952	0.009309	-5.406179
H	0.525326	-0.882591	-0.472023
H	-1.005414	-0.000040	-0.479991
H	0.524315	0.884297	-0.471999
H	-0.906989	-1.559956	3.498738
H	-1.916749	-1.557142	2.051723
H	-0.388012	-2.439536	2.059783
H	1.795272	-0.000542	3.513132
H	2.309150	-0.882423	2.073774
H	2.309197	0.882718	2.074637
H	-0.906108	1.560397	3.499424
H	-0.388174	2.440179	2.060215
H	-1.916765	1.557492	2.053059
H	1.024881	-0.001445	-3.138013
H	-0.521650	0.891524	-3.125149
H	-0.521776	-0.894311	-3.133400

Adduct 11 / (CH3)3-Sn-CH3···H3C-Br / M06-2X

C	0.008669	0.003843	-0.303016
Sn	0.001807	0.001233	1.860165
C	2.037851	0.000334	2.574867
C	-1.019742	1.765006	2.568423
C	-1.019068	-1.765390	2.562387
C	-0.004912	-0.001011	-3.677205
Br	-0.014164	-0.002360	-5.617424
H	0.518707	-0.881799	-0.682928
H	-1.011823	0.006361	-0.686954
H	0.522668	0.887725	-0.681658
H	-1.036432	-1.788793	3.651733
H	-2.047467	-1.781908	2.202265
H	-0.515859	-2.664578	2.207722
H	2.060222	-0.002111	3.664366
H	2.568164	-0.882483	2.218585
H	2.567416	0.885220	2.222589
H	-1.033872	1.786266	3.657861
H	-0.518978	2.665627	2.213958
H	-2.049228	1.780547	2.211409
H	1.027585	-0.004524	-3.347827
H	-0.515460	0.893946	-3.341118
H	-0.522088	-0.891706	-3.339923

Adduct 12 / (CH3)2-Al-CH3···H3C-Br / M06-2X

C	-0.282948	0.482769	-0.016930
Al	1.673522	0.572783	-0.006690
C	2.656916	0.433563	-1.688525
C	2.623999	0.800553	1.684531
C	-3.668494	0.333228	0.036588
Br	-5.606960	0.245166	0.057060
H	-0.702317	0.349027	-1.015559
H	-0.634173	-0.342838	0.609639
H	-0.712510	1.394574	0.409410
H	3.738842	0.494365	-1.564034
H	2.431431	-0.510616	-2.192881
H	2.355637	1.225723	-2.380251
H	3.250019	1.697229	1.660010
H	1.961680	0.879904	2.547620
H	3.303642	-0.038111	1.862028
H	-3.378604	1.178568	-0.576393
H	-3.328777	0.460771	1.057721
H	-3.296006	-0.594158	-0.382444

Adduct 1 / (CH3)3-Si-CH3···H3C-CH3 / MP2

Si	0.00000	0.00000	1.70825
C	0.00000	0.00000	-0.16940
H	-0.00000	1.01909	-0.56600
H	0.88256	-0.50955	-0.56600
H	-0.88256	-0.50955	-0.56600
C	0.00000	-1.77046	2.33492
C	1.53327	0.88523	2.33492
C	-1.53327	0.88523	2.33492
H	1.56140	0.90148	3.42787
H	2.44465	0.39194	1.98616
H	1.56176	1.92116	1.98616
H	-1.56140	0.90148	3.42787
H	-1.56176	1.92116	1.98616
H	-2.44465	0.39194	1.98616
H	-0.00000	-1.80295	3.42787
H	-0.88289	-2.31310	1.98616
H	0.88289	-2.31310	1.98616
C	0.00000	0.00000	-3.98451
C	0.00000	0.00000	-5.50822
H	-0.00000	1.01673	-5.90481
H	0.88051	-0.50836	-5.90481
H	-0.88051	-0.50836	-5.90481
H	-0.88006	0.50810	-3.58650
H	-0.00000	-1.01621	-3.58650
H	0.88006	0.50810	-3.58650

Adduct 2 / (CH3)3-Sn-CH3···H3C-CH3 / MP2

Sn	1.16204	0.00046	-0.00013
C	-0.97062	-0.02893	0.03062

H	-1.35768	-0.54611	-0.84906
H	-1.33214	-0.54470	0.92193
H	-1.36628	0.98820	0.03551
C	1.88555	1.02234	1.72730
C	1.90208	-2.00054	-0.00325
C	1.83537	1.00903	-1.75544
H	2.99329	-2.00232	-0.01751
H	1.56587	-2.53162	0.88886
H	1.54270	-2.53744	-0.88275
H	2.92600	1.03168	-1.78621
H	1.47355	0.49858	-2.64957
H	1.46647	2.03600	-1.76896
H	2.97662	1.04477	1.72666
H	1.51738	2.04954	1.74329
H	1.54938	0.51909	2.63541
C	-4.79267	-0.01952	0.00654
C	-6.31591	0.01510	-0.00504
H	-6.71696	-0.47281	-0.89506
H	-6.73079	-0.49535	0.86579
H	-6.68937	1.04052	0.0051530
H	-4.37582	0.49050	-0.86364
H	-4.38969	0.46804	0.89590
H	-4.41738	-1.04430	-0.00366

Adduct 3 / (CH3)2-Al-CH3···H3C-CH3 / MP2

Al	2.05335	0.00064	0.00255
C	0.08956	-0.05885	0.03639
H	-0.31885	-1.07214	0.04480
H	-0.30421	0.46170	0.91644
H	-0.33418	0.45913	-0.83115
C	2.98421	1.73149	-0.01335
C	3.08840	-1.66997	-0.01543
H	4.16945	-1.51385	-0.03479
H	2.85865	-2.28232	0.86347
H	2.82739	-2.28299	-0.88509
H	3.62367	1.82400	-0.89809
H	2.30957	2.59066	-0.00299
H	3.65150	1.82490	0.85052
C	-3.80266	-0.01518	0.01490
C	-5.32560	0.01149	-0.02626
H	-5.73928	-0.99785	-0.06152
H	-5.73777	0.50574	0.85520
H	-5.68946	0.54742	-0.90462
H	-3.38887	-0.50920	-0.86579
H	-3.38739	0.99341	0.05020
H	-3.43729	-0.55083	0.89270

Adduct 4 / (CH3)3-Si-CH3···H3C-N(CH3)2

C	0.92514	0.01272	-0.06781
Si	2.80143	0.00000	0.01715
C	3.45497	-1.54301	-0.83049
C	3.47661	1.52414	-0.84750

C	3.34571	0.00613	1.81448
C	-2.76622	0.00577	-0.26878
N	-4.20970	0.00000	-0.42051
H	0.50061	-0.86447	0.42846
H	0.51298	0.90100	0.41909
H	0.57610	0.00964	-1.10421
H	4.43595	-0.00138	1.89741
H	2.97882	0.89471	2.33542
H	2.96590	-0.87108	2.34536
H	4.54726	-1.57965	-0.79743
H	3.07725	-2.44877	-0.34831
H	3.15292	-1.57479	-1.88088
H	4.56933	1.54558	-0.81501
H	3.17473	1.54866	-1.89813
H	3.11197	2.44044	-0.37526
H	-2.33887	-0.87690	-0.74807
H	-2.34612	0.89284	-0.74637
H	-2.45809	0.00604	0.79284
C	-4.76851	-1.19187	0.19076
C	-4.77825	1.18611	0.19299
H	-5.86165	1.18845	0.05993
H	-4.56116	1.23945	1.27555
H	-4.37041	2.07977	-0.28289
H	-5.85186	-1.20285	0.05769
H	-4.35336	-2.08126	-0.28679
H	-4.55098	-1.24546	1.27323

Adduct 5 / (CH3)3-Sn-CH3···H3C-N(CH3)2 / MP2

C	-0.09836	0.02565	-0.07731
Sn	2.03340	0.00001	0.00965
C	2.76138	-1.76470	-0.94244
C	2.80552	1.71920	-0.98976
C	2.66070	0.01973	2.04853
C	-3.78995	0.00963	-0.27050
N	-5.23376	0.00002	-0.41972
H	-0.51052	-0.84853	0.42991
H	-0.48835	0.92270	0.40703
H	-0.43701	0.01646	-1.11488
H	3.75011	0.00614	2.11146
H	2.29546	0.91751	2.55007
H	2.27197	-0.85393	2.57442
H	3.85171	-1.79375	-0.90707
H	2.37429	-2.65395	-0.44210
H	2.44740	-1.79010	-1.98729
H	3.89626	1.72148	-0.95494
H	2.49180	1.72422	-2.03498
H	2.44138	2.63124	-0.51381
H	-3.36094	-0.87151	-0.75110
H	-3.37299	0.89809	-0.74821
H	-3.47990	0.01005	0.79053
C	-5.78815	-1.19375	0.19188
C	-5.80438	1.18417	0.19567
H	-6.88804	1.18367	0.06463

H	-5.58540	1.23733	1.27786
H	-5.39982	2.07925	-0.28035
H	-6.87172	-1.20762	0.06083
H	-5.37145	-2.08171	-0.28697
H	-5.56844	-1.24736	1.27390

Adduct 6 / (CH3)2-Al-CH3···H3C-N(CH3)2 / MP2

C	1.28163	0.05266	-0.03639
Al	3.24604	0.00266	0.01361
C	4.26122	1.67810	0.16776
C	4.19050	-1.71737	-0.08940
C	-2.46240	-0.02741	-0.24545
N	-3.90436	-0.07167	-0.40704
H	0.83845	-0.70989	0.61198
H	0.86426	1.02095	0.25073
H	0.92220	-0.16501	-1.04931
H	4.70413	-1.92990	0.85536
H	3.53224	-2.56396	-0.29804
H	4.96947	-1.70144	-0.85857
H	3.96527	2.39331	-0.60707
H	4.05423	2.16932	1.12524
H	5.34281	1.54149	0.09773
H	-2.02310	-0.97342	-0.56690
H	-2.04370	0.77140	-0.86012
H	-2.16504	0.15258	0.80375
C	-4.46033	-1.15006	0.38933
C	-4.48816	1.19463	-0.00458
H	-5.57016	1.16500	-0.14578
H	-4.28260	1.42929	1.05592
H	-4.08207	2.00042	-0.61875
H	-5.54219	-1.19314	0.25045
H	-4.03344	-2.10272	0.07054
H	-4.25351	-1.02053	1.46750

Adduct 7 / (CH3)3-Si-CH3···H3C-O(CH3) / MP2

C	0.39993	0.13252	0.02986
Si	2.27165	-0.02896	-0.00078
C	2.78647	-1.02894	-1.50442
C	3.04385	1.68021	-0.08832
C	2.84800	-0.89983	1.55976
C	-3.24191	0.41312	0.01809
O	-4.64219	0.58944	-0.01928
H	-0.07984	-0.84892	0.08152
H	0.06816	0.71189	0.89606
H	0.03279	0.63623	-0.86873
H	3.93584	-1.00929	1.57225
H	2.56005	-0.34032	2.45396
H	2.41253	-1.89971	1.63837
H	3.87311	-1.14111	-1.55112
H	2.34971	-2.03107	-1.48128
H	2.46219	-0.54598	-2.43024
H	4.13541	1.62003	-0.10785

H	2.72419	2.21278	-0.98816
H	2.75985	2.28700	0.77579
H	-2.88933	-0.15921	-0.84907
H	-2.78640	1.40163	0.00216
H	-2.93105	-0.11148	0.93031
C	-5.30617	-0.65571	-0.00131
H	-6.37586	-0.45601	-0.03168
H	-5.02819	-1.26630	-0.86961
H	-5.06980	-1.21843	0.91056

Adduct 8 / (CH3)3-Sn-CH3···H3C-O(CH3) / MP2

C	-0.52693	0.14874	0.00813
Sn	1.60108	-0.01650	0.00005
C	2.22129	-1.07373	-1.74566
C	2.46227	1.93473	-0.00179
C	2.23429	-1.07610	1.73964
C	-4.17176	0.42621	-0.00273
O	-5.57481	0.58509	0.00290
H	-0.98470	-0.84218	0.00934
H	-0.86234	0.68783	0.89599
H	-0.86892	0.68881	-0.87664
H	3.32140	-1.17055	1.75267
H	1.92127	-0.54758	2.64164
H	1.79908	-2.07674	1.75499
H	3.30823	-1.16870	-1.76657
H	1.78547	-2.07413	-1.75942
H	1.90213	-0.54367	-2.64460
H	3.55159	1.86957	-0.00585
H	2.14465	2.48967	-0.88613
H	2.15117	2.48841	0.88565
H	-3.83610	-0.11469	-0.89639
H	-3.72929	1.42076	-0.00022
H	-3.82964	-0.12187	0.88404
C	-6.22243	-0.66875	0.00024
H	-7.29498	-0.48255	0.00478
H	-5.96058	-1.24879	-0.89365
H	-5.95425	-1.25584	0.88762

Adduct 9 / (CH3)2-Al-CH3···H3C-O(CH3) / MP2

C	0.70639	-0.11142	-0.07460
Al	2.66624	0.02024	0.01840
C	3.74994	-1.60948	0.18871
C	3.53445	1.78098	-0.05926
C	-2.97787	-0.24747	-0.32311
O	-4.38018	-0.31767	-0.47602
H	0.23972	0.42930	0.75650
H	0.32549	0.35524	-0.98976
H	0.33726	-1.13948	-0.04804
H	4.19376	1.85098	-0.93162
H	2.82985	2.61406	-0.11310
H	4.17446	1.94351	0.81465
H	4.82465	-1.41751	0.22699

H	3.48125	-2.16479	1.09409
H	3.56548	-2.29203	-0.64807
H	-2.65775	-0.69873	0.62425
H	-2.53009	-0.79690	-1.14911
H	-2.62612	0.79133	-0.34948
C	-5.03415	0.37600	0.56412
H	-6.10564	0.28721	0.39331
H	-4.78821	-0.05321	1.54365
H	-4.75642	1.43760	0.56946

Adduct 10 / (CH3)3-Si-CH3···H3C-Br / MP2

C	-1.40359	-0.01898	-0.00938
Si	-3.28382	0.00161	0.00003
C	-3.88548	1.11522	1.38642
C	-3.90569	0.65298	-1.64715
C	-3.92242	-1.74294	0.27011
C	2.21750	-0.03101	-0.00007
Br	4.14537	0.00628	0.00001
H	-1.01009	-0.39502	0.93928
H	-1.02180	-0.66070	-0.80856
H	-1.00058	0.98582	-0.16484
H	-5.01552	-1.76568	0.28096
H	-3.58415	-2.41530	-0.52301
H	-3.57235	-2.14933	1.22291
H	-4.97785	1.14927	1.41923
H	-3.53495	0.76028	2.35939
H	-3.52510	2.13925	1.25600
H	-4.99845	0.67752	-1.67453
H	-3.54598	1.66881	-1.83217
H	-3.56682	0.02389	-2.47476
H	1.86629	0.61749	0.79659
H	1.87202	0.32478	-0.96579
H	1.89890	-1.05502	0.16890

Adduct 11 / (CH3)3-Sn-CH3···H3C-Br / MP2

C	-0.32250	-0.02563	-0.01458
Sn	-2.45838	0.00086	0.00001
C	-3.13272	1.50499	1.35296
C	-3.16865	0.42949	-1.96453
C	-3.18022	-1.90566	0.62593
C	3.28815	-0.02872	0.00071
Br	5.21636	0.00586	-0.00015
H	0.05975	-0.24317	0.98452
H	0.04142	-0.79139	-0.70224
H	0.06539	0.94354	-0.33377
H	-4.27136	-1.91257	0.64045
H	-2.83760	-2.68499	-0.05680
H	-2.81863	-2.13880	1.62880
H	-4.22335	1.53496	1.37472
H	-2.77133	1.29884	2.36180
H	-2.76498	2.48544	1.04579
H	-4.25968	0.44694	-1.97859

H	-2.80200	1.40177	-2.29783
H	-2.82495	-0.33105	-2.66760
H	2.93607	0.74178	0.67958
H	2.94565	0.16465	-1.01111
H	2.96755	-1.01090	0.33405

Adduct 12 / (CH3)2-Al-CH3···H3C-Br / MP2

C	1.76660	-0.02187	0.03319
Al	3.73440	0.00075	0.00103
C	4.72922	-1.69243	-0.01481
C	4.68971	1.71662	-0.01421
C	-1.88056	-0.01216	0.02821
Br	-3.80885	0.00246	-0.00933
H	1.34411	-1.02951	0.04560
H	1.38605	0.51054	0.91205
H	1.35798	0.50050	-0.83909
H	5.81356	-1.56115	-0.02997
H	4.48222	-2.30047	0.86234
H	4.45772	-2.29807	-0.88640
H	5.34200	1.79444	-0.89081
H	4.02808	2.58585	-0.01717
H	5.34684	1.80526	0.85790
H	-1.52817	-0.51826	-0.86504
H	-1.53439	1.01643	0.04892
H	-1.56334	-0.54252	0.92064
